# Supplementary material for: Global diversity and biogeography of potential phytopathogenic fungi in a changing world
Source: Nat Commun. 2023 Oct 14;14:6482. doi: 10.1038/s41467-023-42142-4 (PMC10576792; doi:10.1038/s41467-023-42142-4)
Supplement: Supplementary file 1 — Supplementary Information [file 41467_2023_42142_MOESM1_ESM.pdf]

This file includes:

Supplementary Figures 1 to 10;

Supplementary Tables 1 to 5.

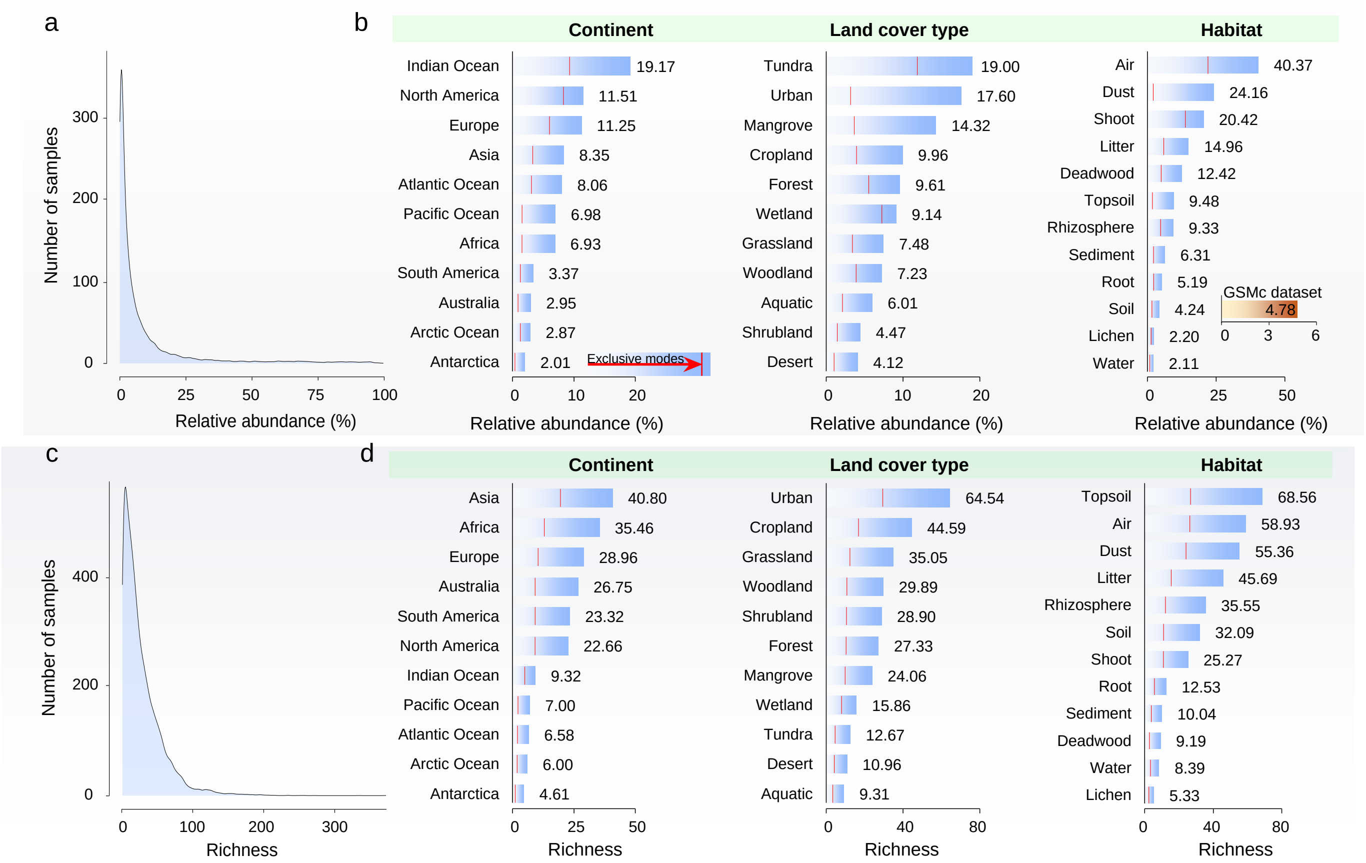

Supplementary Fig. 1 | Relative abundance and diversity of potential phytopathogenic fungi. a, Distribution of the relative abundance of total potential phytopathogenic fungi across the 20,312 samples surveyed. **b,** The mean relative abundance of total potential phytopathogenic fungi in each continent, land cover type, and habitat. The relative abundance of potential phytopathogenic fungi in soils can be further validated against the Global Soil Mycobiome consortium (GSMc) dataset, which shows a very close match to that of our main dataset. **c,** Distribution of the species richness indicated by number of observed potential phytopathogenic species hypotheses (ppSHs; ppSHs can be treated as potential phytopathogenic fungal species) from the communities across the 20,312 samples surveyed. **d,** The mean species richness of potential phytopathogenic fungi in each continent, land cover type, and habitat.

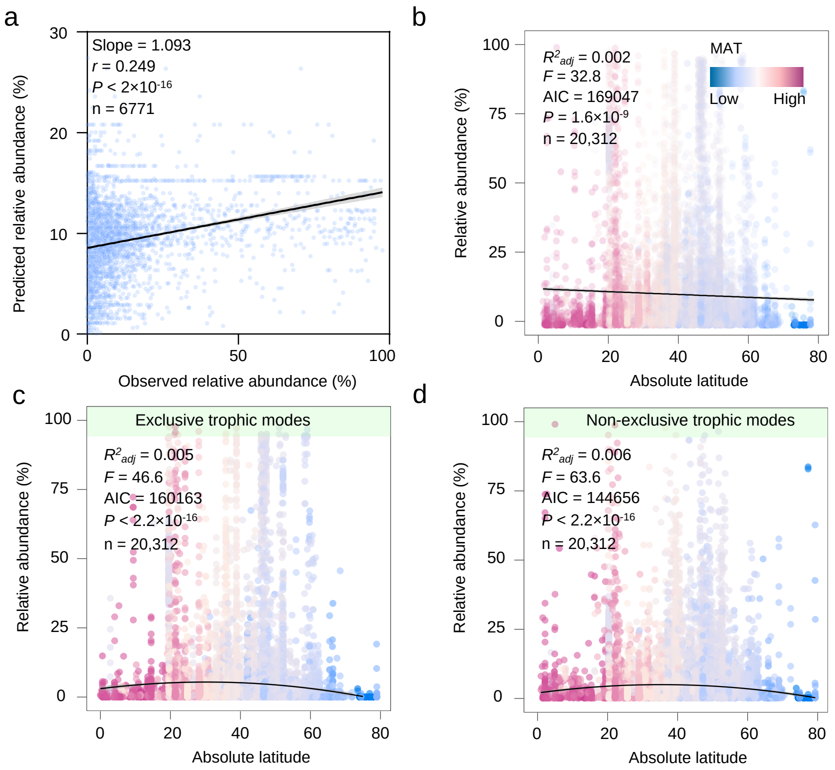

Supplementary Fig. 2 | Relative abundance of potential phytopathogenic fungi and effecting factors. **a,** Cross-validation on the relative abundance-climate model. The whole dataset was divided into two parts with 2/3 samples as modelling dataset and the remaining 1/3 samples as validation dataset. The x-axis shows the observed abundance in the validation dataset, and the y-axis shows the predicted abundance in the validation dataset. Pearson correlation test was conducted to investigate the correlation between observed and predicted abundance. Lines represent the least squares regression fits and shaded areas represent the 95% confidence intervals. We applied one-side *F* and two-side *t* tests, and then calculated *P* values as shown. n represents the number of samples in the validation dataset. **b,** Latitudinal distribution of the relative abundance of potential phytopathogenic fungi, plotting relative abundance of potential phytopathogenic fungi against the absolute latitude of sampling locations. The samples were colored according to the annual mean air temperature (MAT). The line shows the simple linear regression. The R^2 and *F* values of simple linear regression were lower, and AIC and *P* values were higher than that of second-order polynomial regression, demonstrating the poor fitting efficiency of simple linear fitting. n represents the number of independent samples. **c,** Latitudinal distribution of the relative abundance of potential phytopathogenic fungi with exclusive trophic modes (plant pathogens only). n represents the number of independent samples. **d,** Latitudinal distribution of the relative abundance of potential phytopathogenic fungi with non-exclusive trophic modes (plant pathogen and endophyte and/or saprotrophic fungi). n represents the number of independent samples.

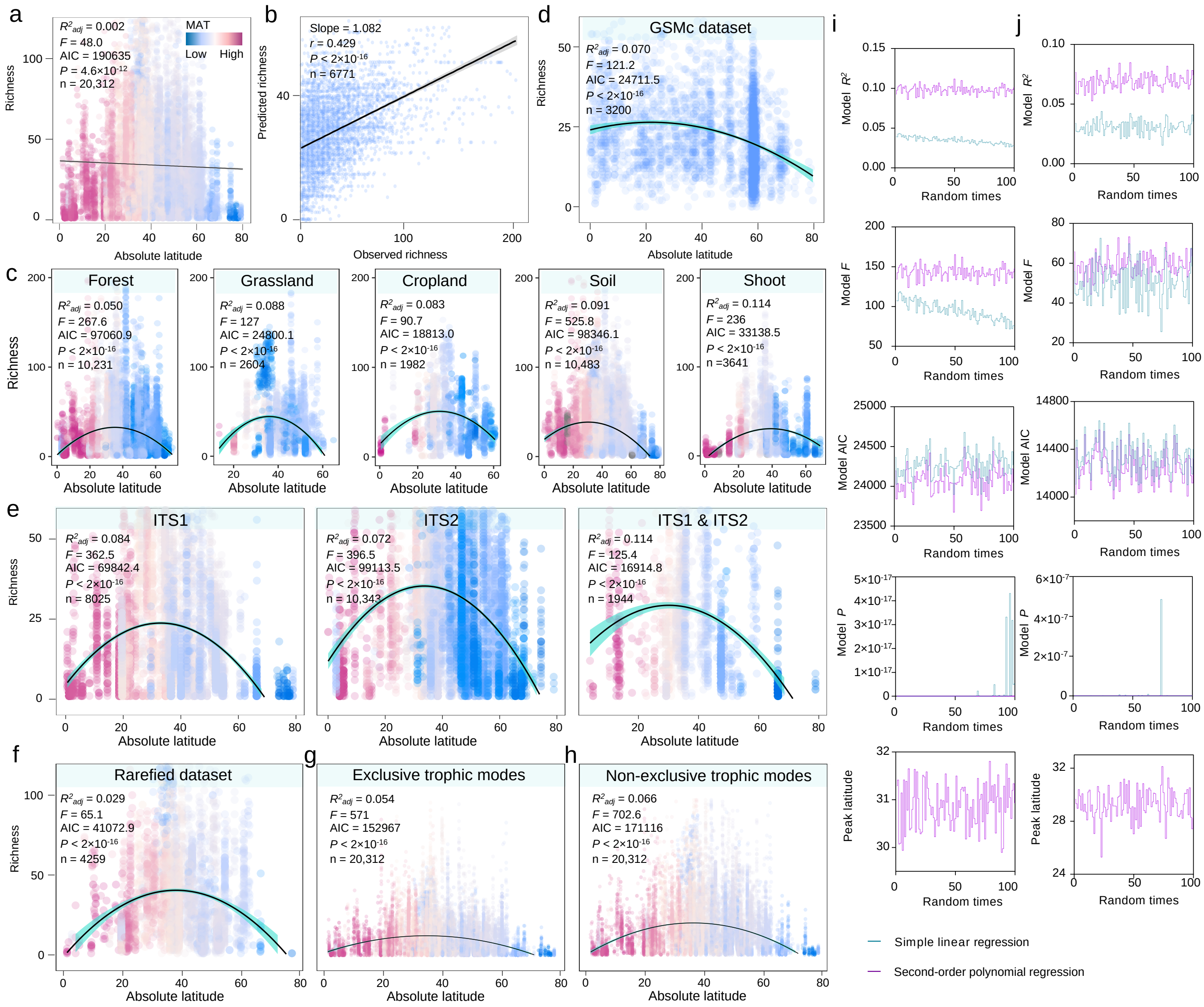

Supplementary Fig. 3 | Alpha diversity and effecting factors. a, Latitudinal distribution of potential phytopathogenic fungal diversity, plotting richness of phytopathogenic fungi against the absolute latitude of sampling locations. The samples were colored according to the annual mean air temperature (MAT). The line shows the simple linear regression. The R^2 and F values of simple linear regression were lower, and AIC and P values were higher than that of second-order polynomial regression, demonstrating the poor fitting efficiency of simple linear fitting. **b,** Cross-validation on the diversity-climate model. The whole dataset was divided into two parts with 2/3 samples as modelling dataset and the remaining 1/3 samples as validation dataset. The x-axis shows the observed diversity in the validation dataset, and the y-axis shows the predicted diversity in the validation dataset. Pearson correlation test was conducted to investigate the correlation between observed and predicted diversity. Lines represent the least squares regression fits and shaded areas represent the 95% confidence intervals. We applied one-side F and two-side t tests, and then calculated P values as shown. **c,** Latitudinal distribution of potential phytopathogenic fungal diversity in main land cover types and habitats. Colors represent the annual mean air temperatures (MAT) of sampled locations. The line shows the second-order polynomial fit based on ordinary least squares regression, confirming a peak in phytopathogenic fungal species richness at intermediate latitudes in different main land cover types and habitats. Shaded areas represent the 95% confidence intervals. **d,** Latitudinal distribution of potential phytopathogenic fungal diversity in the Global Soil Mycobiome consortium (GSMc) dataset. Since the data in GSMc were *de novo* picked, we re-assigned the fungal OTUs based on UNITE database to get the SH IDs of each OTU. After assigning the OTUs to our referenced phytopathogenic fungi database, the richness of potential

phytopathogenic fungi was plotted against the absolute latitude. **e,** The richness-latitude relationship of samples that sequenced different ITS regions. Colors represent the annual mean air temperatures (MAT) of sampled locations. The line shows the second-order polynomial fit based on ordinary least squares regression, confirming a peak in phytopathogenic fungal species richness at intermediate latitudes. Shaded areas represent the 95% confidence intervals. **f,** The richness-latitude relationship of samples with equal phytopathogenic fungal sequences. After rarefying the ppSH sequences to even depth (4000 sequences per sample), we determined the richness-latitude relationship, and confirmed that our finding on the richness-latitude relationship is not biased by the sequencing depth. **g,** Latitudinal distribution of the richness of potential phytopathogenic fungi with exclusive trophic modes (plant pathogens only). **h,** Latitudinal distribution of the richness of potential phytopathogenic fungi with non-exclusive trophic modes (plant pathogen and endophyte and/or saprotrophic fungi). n values in a-h represent the number of independent samples. **i,** Model parameters comparison between simple linear regression and second-order polynomial regression on fitting diversity-latitude relationship of resampling dataset which randomly selected **300 samples** for in North America, Europe, Asia, Australia, South America, Antarctica and Africa where have >300 samples. **j,** Model parameters comparison between simple linear regression and second-order polynomial regression on fitting diversity-latitude relationship of resampling dataset which randomly selected **150 samples** for in North America, Europe, Asia, Australia, South America, Antarctica and Africa. Both random resampling procedures (300/150 samples) were conducted for 100 times (x-axis). Generally, higher model R^2 and F values, and lower AIC and P values indicate better fitting efficiency. Based on these, our comparison consistently showed a better fitting efficiency of second-order polynomial regression than simple liner regression. Then, the peak latitude (the absolute latitude where the potential phytopathogenic fungal diversity peaks) was calculated based on the fitting parameters of second-order polynomial regression following the formula: peak latitude = $-\beta/(2\alpha)$, where α and β are the second-order and first-order coefficients, respectively.

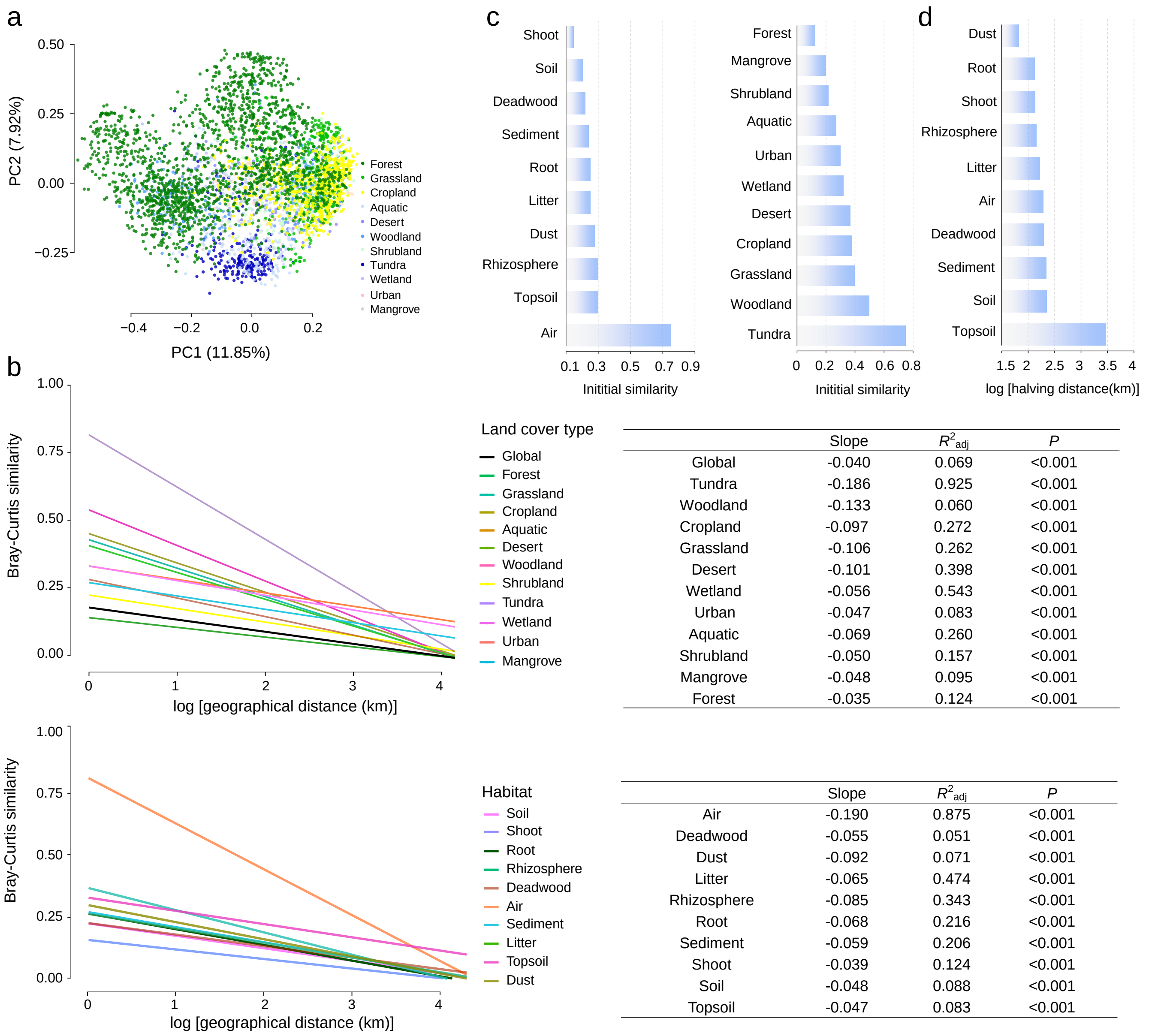

	Slope	R^2_{adj}	*P*
Global	-0.040	0.069	<0.001
Tundra	-0.186	0.925	<0.001
Woodland	-0.133	0.060	<0.001
Cropland	-0.097	0.272	<0.001
Grassland	-0.106	0.262	<0.001
Desert	-0.101	0.398	<0.001
Wetland	-0.056	0.543	<0.001
Urban	-0.047	0.083	<0.001
Aquatic	-0.069	0.260	<0.001
Shrubland	-0.050	0.157	<0.001
Mangrove	-0.048	0.095	<0.001
Forest	-0.035	0.124	<0.001

	Slope	R^2_{adj}	*P*
Air	-0.190	0.875	<0.001
Deadwood	-0.055	0.051	<0.001
Dust	-0.092	0.071	<0.001
Litter	-0.065	0.474	<0.001
Rhizosphere	-0.085	0.343	<0.001
Root	-0.068	0.216	<0.001
Sediment	-0.059	0.206	<0.001
Shoot	-0.039	0.124	<0.001
Soil	-0.048	0.088	<0.001
Topsoil	-0.047	0.083	<0.001

Supplementary Fig. 4 | Beta diversity of potential phytopathogenic fungal communities. a, Principal co-ordinates analysis (PCoA) on potential phytopathogenic fungal community based on Sørensen distance. Samples were colored by different land cover types. **b,** Distance-decay relationships of potential phytopathogenic fungal communities in different land cover types and different habitats. The Bray-Curtis similarity was used to indicate community similarity, and the geographical distance was log-transformed. The right panel shows the DDR slope, and the slope significance was tested using one-side *F* and two-side *t* tests, and then calculated *P* values as shown. **c,** Initial similarity of potential phytopathogenic fungal communities in different land cover types and different habitats. The initial similarity represents the community similarity (Bray-Curtis similarity) within one kilometer in the current study (see Methods). **d,** Halving distance of potential phytopathogenic fungal communities in different habitats. Halving distance represents the distance at which community similarity halves (see Methods). Habitats lichen and freshwater were not analyzed due to their low ppSH reads number.

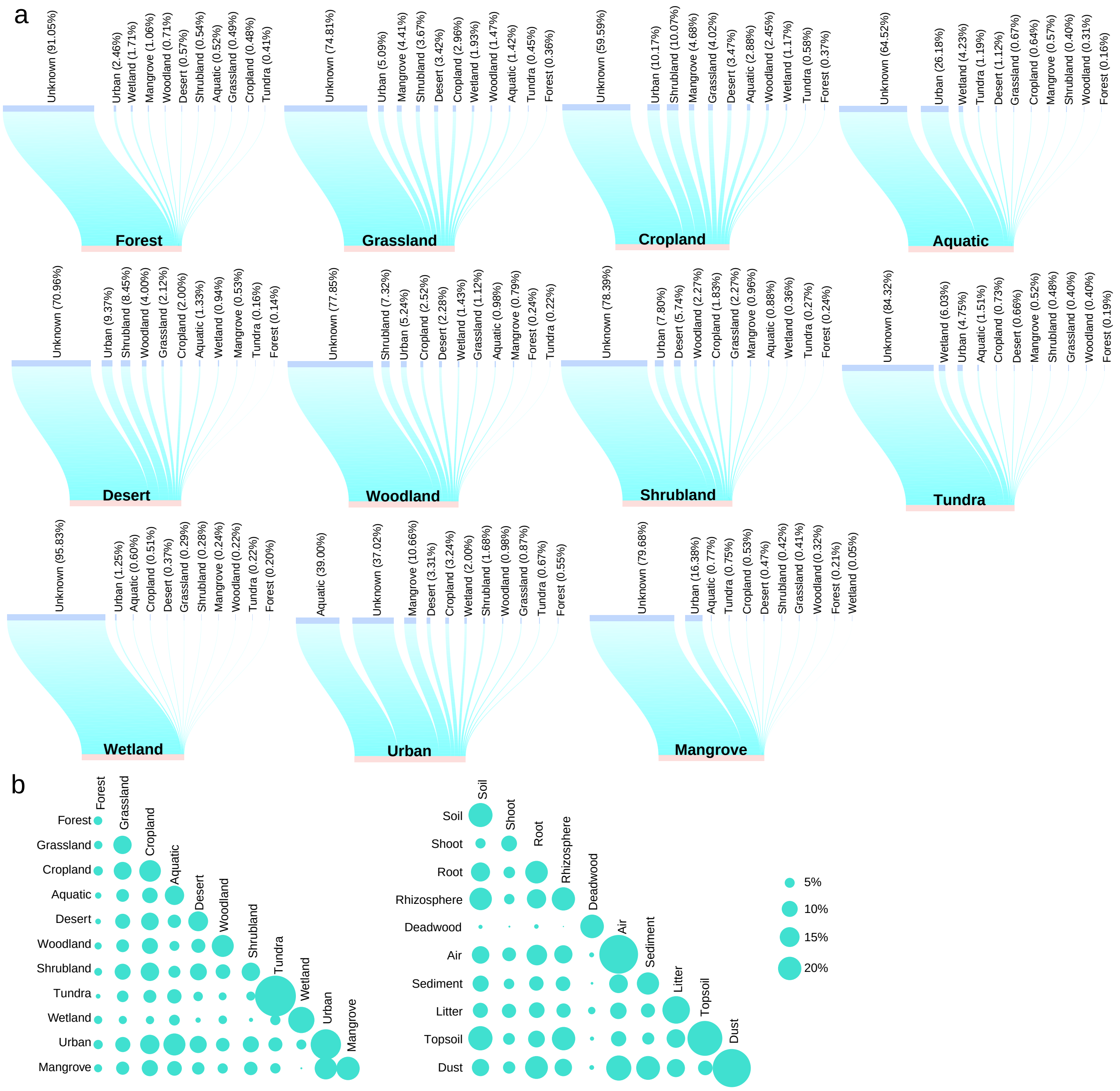

Supplementary Fig. 5 | Potential community dynamics between different land cover types or different habitats. a, Source tracking of potential phytopathogenic fungi in land cover types. To determine where the potential phytopathogenic fungi in different land cover types come from, we estimated the source of potential phytopathogenic fungi at genus level in different land cover types using SourceTracker. The numbers in the brackets reflect the proportions of potential phytopathogenic fungi that derived from different land cover types. The results showed that most potential phytopathogenic fungi cannot be source-tracked (as indicated by 'Unknown'). **b,** The proportion of shared potential phytopathogenic fungal sequences between different land cover types and between different habitats. The circle size reflects the proportion of shared sequences between different land cover types or different habitats. Habitats lichen and freshwater were not analyzed due to their low ppSH reads number.

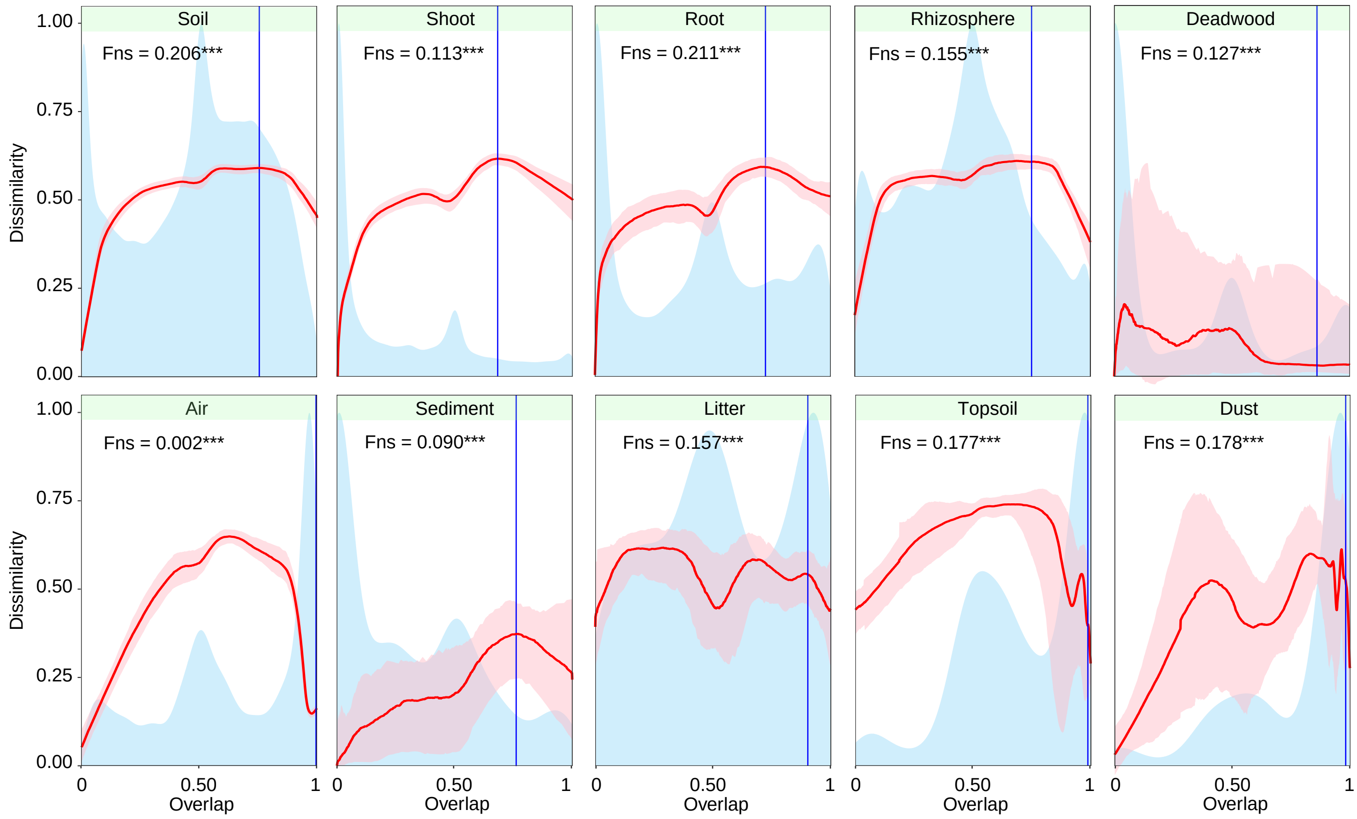

Supplementary Fig. 6 | Universal ecological dynamics of potential phytopathogenic fungi in different habitats. The ecological universality of potential phytopathogenic fungi was assessed using dissimilarity-overlap curves (DOC). For DOCs, the dissimilarity-overlap curve is in red, the distribution density of sample pair overlap is in light blue, and the point at which a negative DOC is first observed is marked by a vertical blue line (chosen by median of 1000 bootstraps). The fraction of negative slope (Fns) is simply the fraction of data points in the interval where the DOC has a negative slope and significance is as follows: * $P < 0.05$, ** $P < 0.01$, and *** $P < 0.001$. A higher Fns value indicates higher ecological universality (host-independent) of the metacommunity. Habitats lichen and freshwater were not analyzed due to their low ppSH reads number.

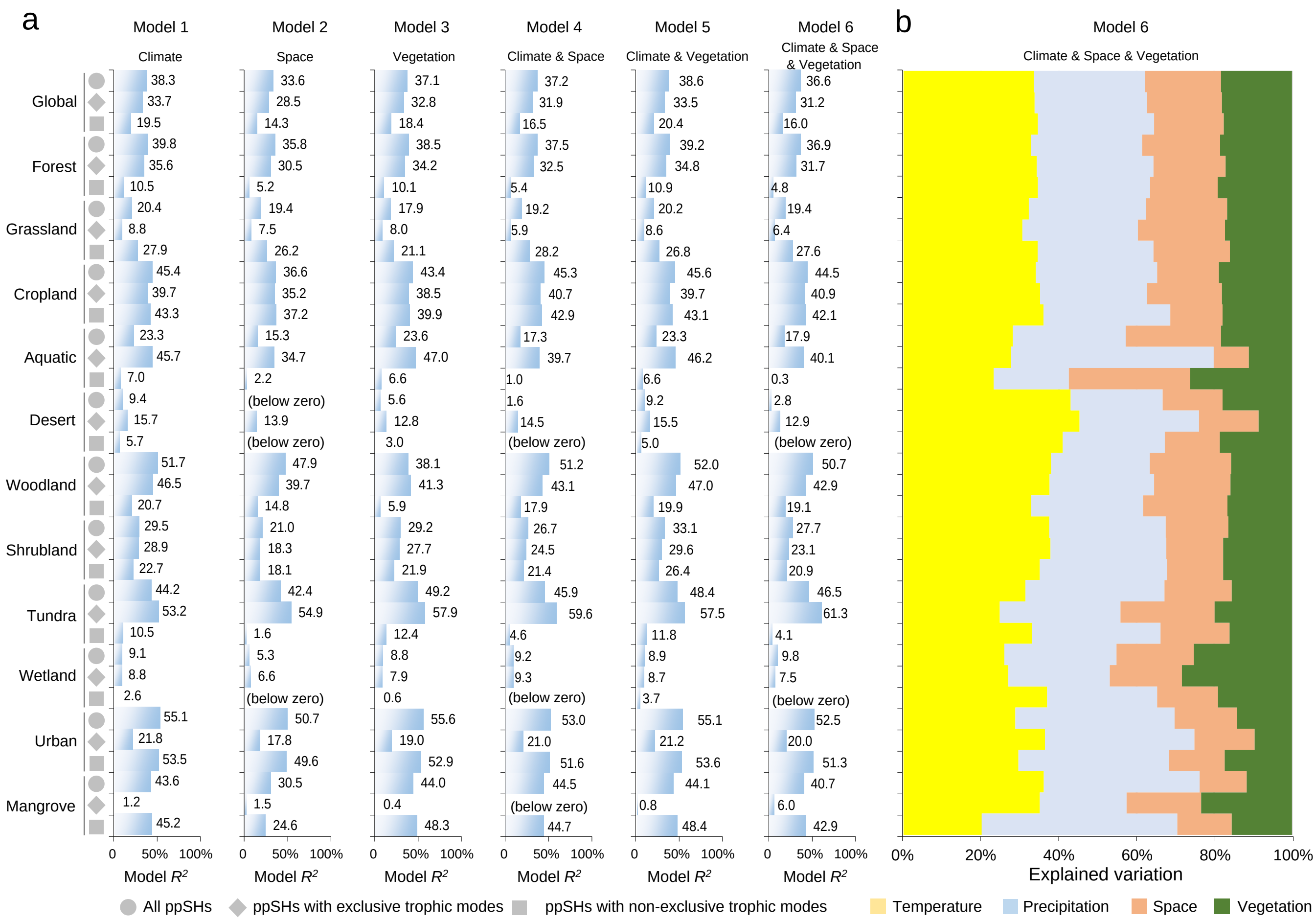

Supplementary Fig. 7 | Factors affecting the relative abundance of potential phytopathogenic fungi. **a,** Random forest model performance for relative abundance of potential phytopathogenic fungi. **b,** Contribution of climatic, spatial and vegetation variable categories to the variation explained by the complete random forest model for relative abundance of potential phytopathogenic fungi. Each tree was fitted based on a random sample of two-third of the observations ("in-bag"), and each tree split was based on a different random subset of one-third of the predictors, while the results were cross-validated against the remaining observations ("out-of-bag"), which is in line with standard protocols. The model performance was assessed based on model R^2 with 999 permutations. Vegetation is indicated by gross primary production and plant diversity.

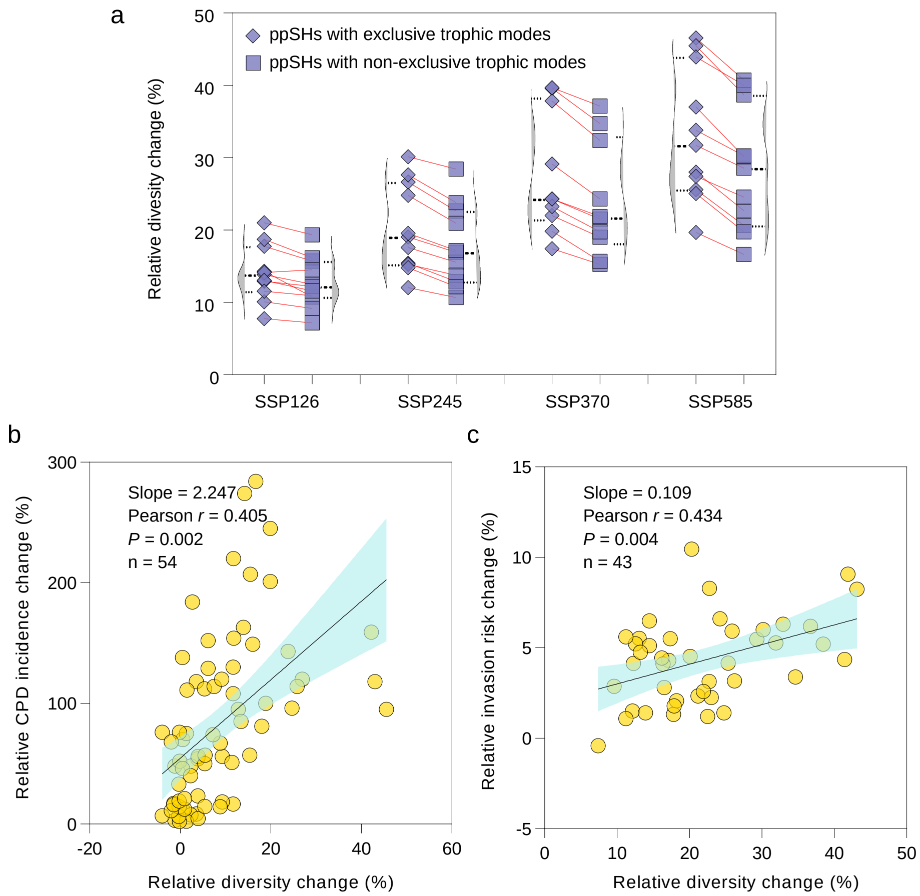

Supplementary Fig. 8 | The predicted change in diversity of potential phytopathogenic fungi under different future climate scenarios. a, The predicted change in diversity of potential phytopathogenic fungi with exclusive trophic modes (plant pathogens only) and non-exclusive trophic modes (plant pathogen and endophyte and/or saprotrophic fungi) under different future climate scenarios. Plot axis labels reflect, shared socioeconomic pathway (SSP); sustainability (SSP126); middle of the road (SSP245); regional rivalry (SSP370); and fossil-fuelled development (SSP585) scenarios. The y-axis shows the relative change in ppSH richness across all samples. **b,** The relationship between changes in potential phytopathogenic fungal diversity and occurrence of crop pests and diseases (CPD). The x-axis shows the average change of potential phytopathogenic fungal diversity in cropland ecosystem. The future CPD data in y-axis were derived from a recently published study, which predicted the future CPD incidence in 2100 under SSP126 and SSP585 scenarios in cropland in China (The raw data in the Figure 5 of that published study were used). We then re-predicted the future diversity of potential phytopathogenic fungi using the samples collected in cropland in China to match the study of CPD incidence, and extracted the diversity changes under two climate scenarios at province level to plot against the CPD incidence. n represents the number of provinces under two climate scenarios. **c,** The relationship between relative change in potential phytopathogenic fungal diversity and relative change in invasion risk. Lines represent the least squares regression fits and shaded areas represent the 95% confidence intervals. We applied one-side *F* and two-side *t* tests, and then calculated *P* values as shown. n represents the number of GCMs under four climate scenarios.

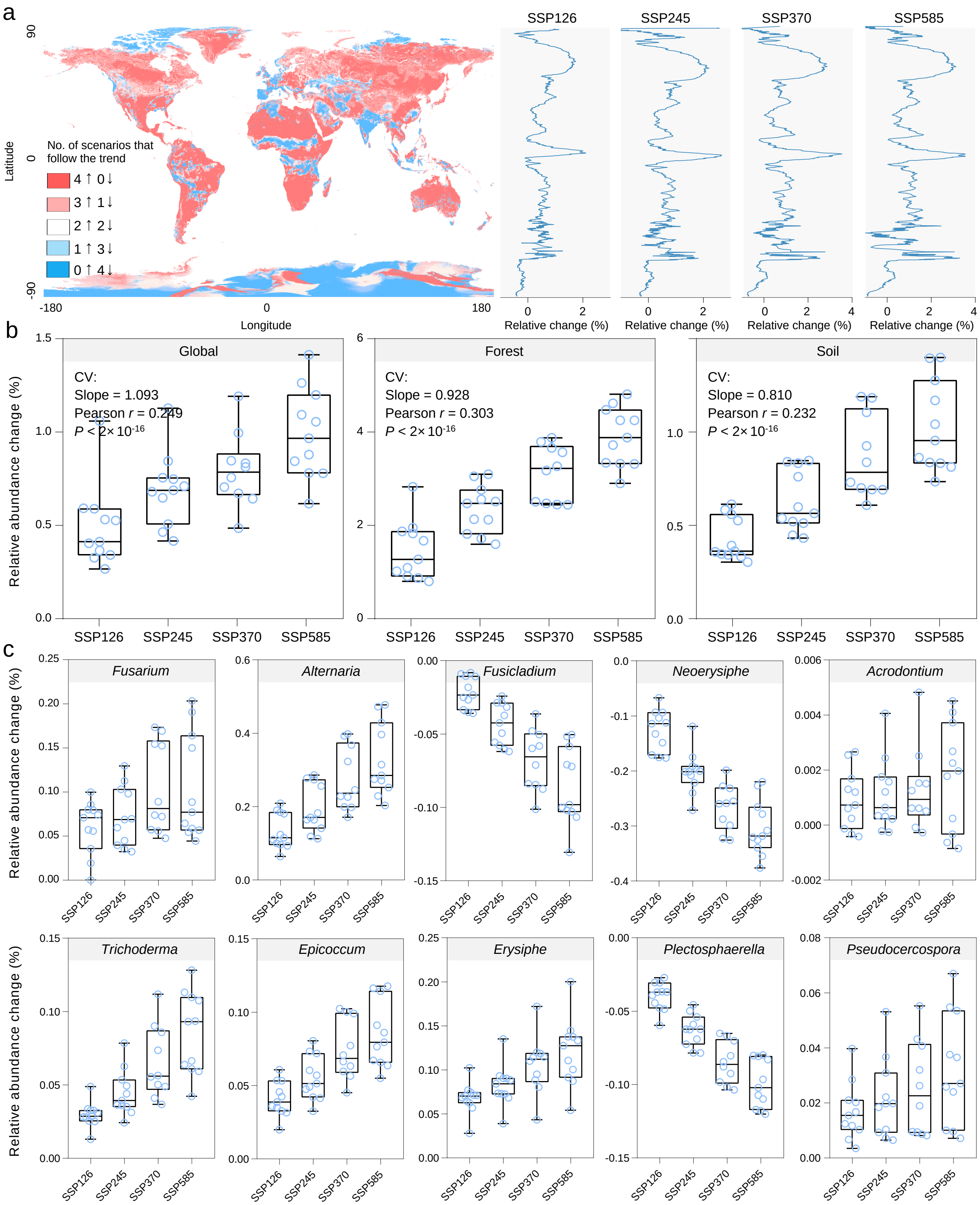

Supplementary Fig. 9 | Relative abundance of potential phytopathogenic fungi by 2100 under future climate change scenarios. a, Predicted change in relative abundance of potential phytopathogenic fungi under future climate-change scenarios. **b,** Predicted change in relative abundance of potential phytopathogenic fungi across all samples and in forest ecosystem and soil habitat. **c,** Predicted change in relative abundance of top ten dominant potential phytopathogenic fungal genera. An abundance-climate model was constructed by GLMs using relative abundance of phytopathogenic fungi and 19 climate variables. This model was used to predict relative abundance of potential phytopathogenic fungi across all land cover

types, or within forest ecosystem, or from soil habitat, under four different climate scenarios. Predictive models were cross-validated (CV) by common Pearson correlation test using 2/3 samples as a model training dataset and 1/3 as a validation dataset. All climate variables were derived from WorldClim2 using a 5 min (~10 km) resolution. The future climate data were derived from eleven different CMIP6 downscaled global change models (GCMs; See detailed information in Methods. NB – The climate data of model FIO-ESM-2-0 under the SSP370 scenario in 2080-2100 are not available). The relative change in relative abundance of potential phytopathogenic fungi under different GCMs compared to current climate conditions were averaged. Plot axis labels reflect, shared socioeconomic pathway (SSP); sustainability (SSP126); middle of the road (SSP245); regional rivalry (SSP370); and fossil-fuelled development (SSP585) scenarios. Box plots indicate median (middle line) with 25th, and 75th percentile (box), and 5th and 95th percentile (whiskers).

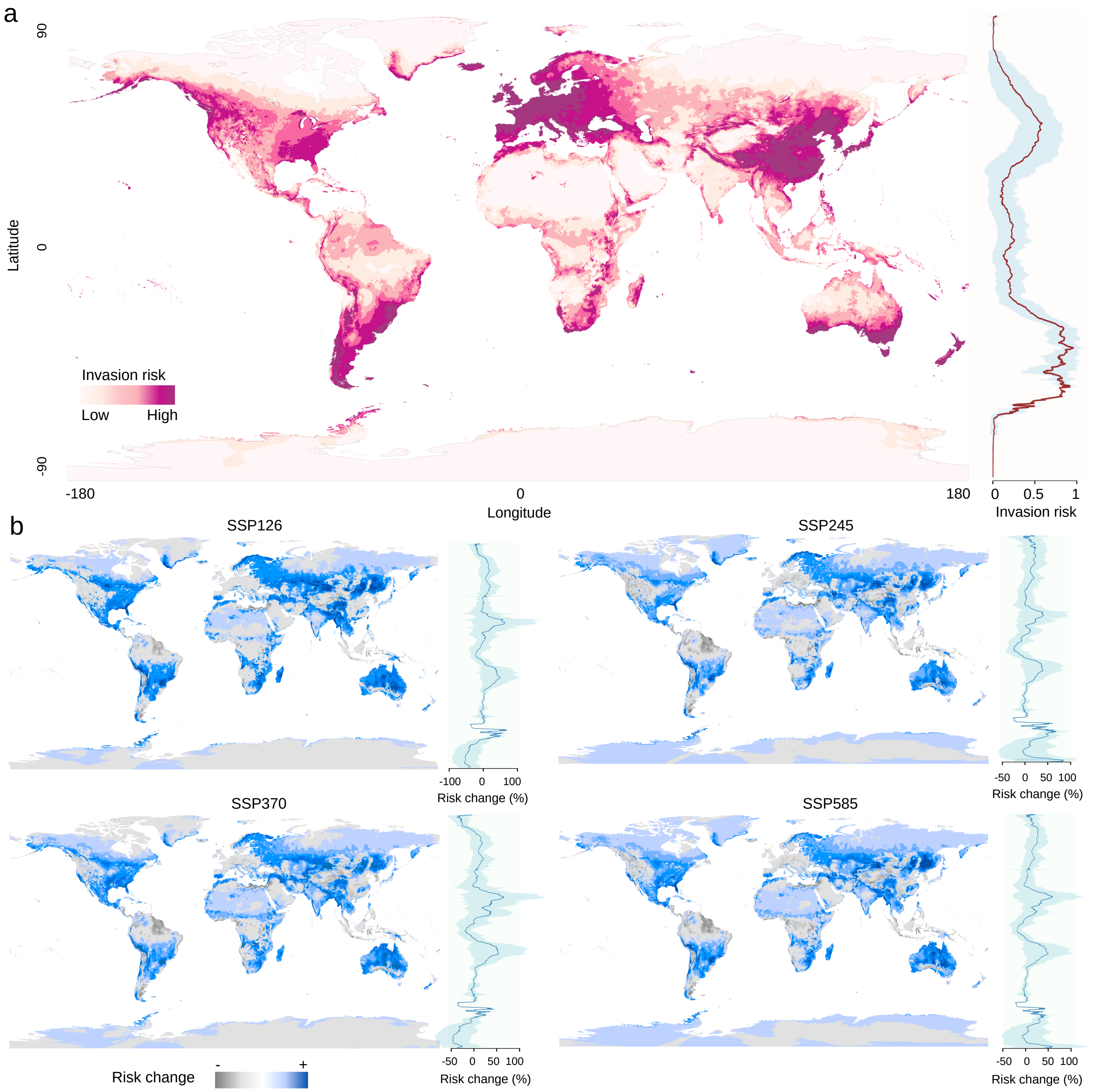

Supplementary Fig. 10 | Global invasion risk of potential phytopathogenic fungi under different future climate scenarios. a, Predicted global invasive risk of potential phytopathogenic fungi under current climate conditions. The invasive risk of phytopathogenic fungi was predicted by a maximum entropy model using current climate data with 30% of the samples as the random seed. The right panel represents the mean invasive risk of phytopathogenic fungi and shaded areas represent the standard deviation at different latitudes. **b**, Relative change in global risks of invasion from potential phytopathogenic fungi under future climate-change scenarios. Future invasion risks were predicted using the climate data derived from eleven different CMIP6 downscaled GCMs, and the change in relative invasion risk under different GCMs compared to current climate conditions were averaged and visualized. "+" and "-" represent increased and decreased invasion risk, respectively. The right panel represents the mean relative change in the risk of invasion from potential phytopathogenic fungi at different latitudes. SSP, shared socioeconomic pathway; SSP126: sustainability; SSP245: middle of the road; SSP370: regional rivalry; SSP585: fossil-fuelled development.

Supplementary Table 1 | Three-way PERMANOVA investigating the effects of continent, land cover type and habitat on potentially phytopathogenic fungal community composition based on Bray-Curtis dissimilarity and Sørensen distance.

	Df	Sums of Sqs	Mean Sqs	*F*	R^2	*P*
Bray-Curtis dissimilarity						
Continent	8	89.790	11.224	35.614	0.046	0.001
Land cover type	10	176.350	17.635	55.956	0.091	0.001
Habitat	9	87.600	9.734	30.885	0.045	0.001
Continent* Land cover type	31	160.860	5.189	16.465	0.083	0.001
Continent*Habitat	22	53.090	2.413	7.657	0.027	0.001
Land cover type*Habitat	20	36.250	1.813	5.751	0.019	0.001
Continent*Land cover type *Habitat	11	24.520	2.230	7.074	0.013	0.001
Residuals	4284	1319.590	0.315	0.677		
Total	4395	1948.060	1.000			
Sørensen distance						
Continent	8	122.690	15.336	79.995	0.094	0.001
Land cover type	10	138.680	13.868	72.337	0.107	0.001
Habitat	9	92.660	10.296	53.704	0.071	0.001
Continent*Land cover type	31	106.060	3.421	17.846	0.081	0.001
Continent*Habitat	22	43.420	1.974	10.295	0.033	0.001
Land cover type*Habitat	20	26.840	1.342	7.001	0.021	0.001
Continent*Land cover type *Habitat	11	16.520	1.501	7.831	0.013	0.001
Residuals	4284	754.780	0.192	0.580		
Total	4395	1301.650	1.000			

Supplementary Table 2 | ROC analysis assessing the projection efficiency on invasion risk of potential phytopathogenic fungi. The data are area under the curve (AUC) values. The AUC ranges from 0 to1, and a higher AUC indicates a stronger projection efficiency. The climate data of model FIO-ESM-2-0 under the SSP370 scenario in 2080-2100 are not available. CMIP6 GCMs: CMIP6 downscaled global change models. SSP, shared socioeconomic pathway; SSP126: sustainability; SSP245: middle of the road; SSP370: regional rivalry; SSP585: fossil-fuelled development.

CMIP6 GCMs	SSP126	SSP245	SSP370	SSP585
ACCESS-ESM1-5	0.899	0.896	0.899	0.896
CanESM5	0.897	0.894	0.895	0.895
CanESM5-CanOE	0.896	0.896	0.894	0.892
CNRM-CM6-1-HR	0.897	0.898	0.894	0.894
CNRM-ESM2-1	0.895	0.898	0.889	0.902
EC-Earth3-Veg	0.898	0.895	0.899	0.898
FIO-ESM-2-0	0.896	0.898	-	0.898
GISS-E2-1-G	0.895	0.898	0.904	0.899
MIROC6	0.900	0.895	0.898	0.901
MRI-ESM2-0	0.896	0.897	0.896	0.899
UKESM1-0-LL	0.896	0.896	0.895	0.891

Supplementary Table 3 | The detailed information about the bioclimatic variables.

Variable number	Variable
BIO1	Annual mean temperature
BIO2	Mean diurnal range (Mean of monthly (max temp - min temp))
BIO3	Isothermality (BIO2/BIO7) (×100)
BIO4	Temperature seasonality (standard deviation ×100)
BIO5	Max temperature of warmest month
BIO6	Min temperature of coldest month
BIO7	Temperature annual range (BIO5-BIO6)
BIO8	Mean temperature of wettest quarter
BIO9	Mean temperature of driest quarter
BIO10	Mean temperature of warmest quarter
BIO11	Mean temperature of coldest quarter
BIO12	Annual precipitation
BIO13	Precipitation of wettest month
BIO14	Precipitation of driest month
BIO15	Precipitation seasonality (coefficient of variation)
BIO16	Precipitation of wettest quarter
BIO17	Precipitation of driest quarter
BIO18	Precipitation of warmest quarter
BIO19	Precipitation of coldest quarter

Supplementary Table 4 | The variation inflation factor values of predicting factors.

PCNM: principal coordinates of neighbor matrices.

Variable	Variation inflation factor
BIO2	1.577960
BIO3	4.774115
BIO4	9.849872
BIO8	2.105976
BIO9	3.404001
BIO13	7.732439
BIO14	7.012767
BIO15	4.912940
BIO18	6.584798
BIO19	6.562696
Gross primary production	1.251985
Plant diversity	2.240571
Absolute latitude	7.234072
Longitude	1.477154
Standardized PCNM	1.001316

Supplementary Table 5 | Parameters of the formula for predicting future changes in diversity and relative abundance of potential phytopathogenic fungi. All listed variables were significant under $P < 0.05$. '-' indicates discarded variable.

Variable	Diversity							Relative abundance		
	Global	Forest	Grassland	Cropland	Soil	Shoot	Root	Global	Forest	Soil
BIO1	-4.407	1.938	-17.560	-17.560	0.670	-4.750	-1.275	-2.097	-5.856	-
BIO2	1.673	2.900	8.474	8.474	0.818	3.430	-	0.529	0.896	-
BIO3	-0.632	-1.012	-2.150	-2.150	-0.500	-1.139	-	-0.092	-0.296	-0.058
BIO4	-0.398	-0.420	0.095	0.095	-	-0.695	-	0.023	0.150	-
BIO5	-	-	-	-	-	-	-	-	-	-
BIO6	-	-	-	-	-	-	-	-	-	-
BIO7	-	-	-	-	-	-	-	-	-	-
BIO8	0.590	1.463	0.528	0.528	-	-	1.371	0.180	0.337	0.109
BIO9	0.297	0.788	-1.358	-1.358	-0.942	1.329	0.806	-0.384	-0.703	-
BIO10	17.373	14.212	-	-	-	29.516	-	-	-3.171	0.036
BIO11	-13.205	-17.320	17.633	17.633	1.249	-25.340	-	2.468	10.074	-
BIO12	-	-	-	-	-	-0.040	-	-	-	0.003
BIO13	0.266	-0.065	1.462	1.462	0.181	0.127	-	0.067	0.070	0.040
BIO14	-	0.456	-	-	-0.382	-	0.523	-0.501	0.134	-
BIO15	0.233	0.389	-	-	0.302	0.127	-0.156	0.019	-	0.037
BIO16	-0.139	-	-0.558	-0.558	-0.100	-	-0.023	-0.039	-0.04	-0.025
BIO17	-	-0.071	-	-	0.175	0.111	-0.234	0.177	-	-
BIO18	0.039	-	-	-	-	0.049	0.014	0.004	-	-
BIO19	-	-	-	-	-	-	0.015	-	0.006	-
Intercept	39.987	32.211	124.656	124.656	32.368	38.098	24.280	7.379	8.172	3.672
