# Supplementary material for: Global diversity and biogeography of potential phytopathogenic fungi in a changing world
Source: Nat Commun. 2023 Oct 14;14:6482. doi: 10.1038/s41467-023-42142-4 (PMC10576792; doi:10.1038/s41467-023-42142-4)
Supplement: Supplementary file 2 — Description of Additional Supplementary Files [file 41467_2023_42142_MOESM2_ESM.pdf]

Description of Additional Supplementary Files

Supplementary Data 1: Top ten abundant and frequent (relative) potential phytopathogenic fungal genera in different continents/oceanic island regions.

Supplementary Data 2: Top ten abundant and frequent (relative) potential phytopathogenic fungal genera in different land cover types.

Supplementary Data 3: Top ten abundant and frequent (relative) potential phytopathogenic fungal genera in different habitats.
