# Supplementary material for: Global diversity and biogeography of potential phytopathogenic fungi in a changing world
Source: Nat Commun. 2023 Oct 14;14:6482. doi: 10.1038/s41467-023-42142-4 (PMC10576792; doi:10.1038/s41467-023-42142-4)
Supplement: Supplementary file 6 — Reporting Summary [file 41467_2023_42142_MOESM6_ESM.pdf]

nature portfolio

Corresponding author(s): Baozhan Wang, Alex J. Dumbrell, Jiandong Jiang

Last updated by author(s): Sep 10, 2023

Reporting Summary

Nature Portfolio wishes to improve the reproducibility of the work that we publish. This form provides structure for consistency and transparency in reporting. For further information on Nature Portfolio policies, see our Editorial Policies and the Editorial Policy Checklist.

Statistics

For all statistical analyses, confirm that the following items are present in the figure legend, table legend, main text, or Methods section.

n/a	Confirmed	
☐	☒	The exact sample size (*n*) for each experimental group/condition, given as a discrete number and unit of measurement
☐	☒	A statement on whether measurements were taken from distinct samples or whether the same sample was measured repeatedly
☐	☒	The statistical test(s) used AND whether they are one- or two-sided<br>*Only common tests should be described solely by name; describe more complex techniques in the Methods section.*
☐	☒	A description of all covariates tested
☐	☒	A description of any assumptions or corrections, such as tests of normality and adjustment for multiple comparisons
☐	☒	A full description of the statistical parameters including central tendency (e.g. means) or other basic estimates (e.g. regression coefficient) AND variation (e.g. standard deviation) or associated estimates of uncertainty (e.g. confidence intervals)
☐	☒	For null hypothesis testing, the test statistic (e.g. *F*, *t*, *r*) with confidence intervals, effect sizes, degrees of freedom and *P* value noted<br>*Give P values as exact values whenever suitable.*
☒	☐	For Bayesian analysis, information on the choice of priors and Markov chain Monte Carlo settings
☒	☐	For hierarchical and complex designs, identification of the appropriate level for tests and full reporting of outcomes
☐	☒	Estimates of effect sizes (e.g. Cohen's *d*, Pearson's *r*), indicating how they were calculated

Our web collection on statistics for biologists contains articles on many of the points above.

Software and code

Policy information about availability of computer code

Data collection	All data were manually collected. A total of 20312 samples were collected in this study, of which 643 were newly generated and 19669 were derived from GlobalFungi database (https://globalfungi.com/). Data collection information is included in the method section of our manuscript.
Data analysis	A closed-reference method was conducted to analyze our newly generated sequencing data. Data analyses were conducted in R and ArcGis. Data analysis information is included in the method section of our manuscript. The PERMANOVA was conducted using R package 'vegan'. The principal coordinates of neighbor matrices (PCNM) algorithm was determined using R package 'spacemakeR'. Multivariate negative binomial General Linear Models were conducted using R package 'mvabund'. Random forest model was constructed using the R packages 'randomForest' and 'rfUtilities'. Used codes are available in Figshare (https://doi.org/10.6084/m9.figshare.20324634).

For manuscripts utilizing custom algorithms or software that are central to the research but not yet described in published literature, software must be made available to editors and reviewers. We strongly encourage code deposition in a community repository (e.g. GitHub). See the Nature Portfolio guidelines for submitting code & software for further information.

Data

Policy information about availability of data

All manuscripts must include a data availability statement. This statement should provide the following information, where applicable:

- Accession codes, unique identifiers, or web links for publicly available datasets
- A description of any restrictions on data availability
- For clinical datasets or third party data, please ensure that the statement adheres to our policy

All raw data used in the current study including reference database, sample metadata, climate data and species-abundance dataset are publicly available in Figshare (https://doi.org/10.6084/m9.figshare.20324634). The UNITE fungal database is available in https://unite.ut.ee/. The samples and the corresponding metadata in GlobalFungi database are available in https://globalfungi.com/. The current and future climate data are available in WorldClim2 (https://www.worldclim.org/).

Human research participants

Policy information about studies involving human research participants and Sex and Gender in Research.

Reporting on sex and gender	This information has not been collected.
Population characteristics	This information has not been collected.
Recruitment	This information has not been collected.
Ethics oversight	This information has not been collected.

Note that full information on the approval of the study protocol must also be provided in the manuscript.

Field-specific reporting

Please select the one below that is the best fit for your research. If you are not sure, read the appropriate sections before making your selection.

☐ Life sciences ☐ Behavioural & social sciences ☒ Ecological, evolutionary & environmental sciences

For a reference copy of the document with all sections, see nature.com/documents/nr-reporting-summary-flat.pdf

Ecological, evolutionary & environmental sciences study design

All studies must disclose on these points even when the disclosure is negative.

Study description	Here, we use a novel global dataset that combined newly generated DNA sequence data with previously published mycobiome sequences from the GlobalFungi database to study the global diversity and biogeography of phytopathogenic fungi.
Research sample	To build a global atlas of phytopathogenic fungi, the samples distributed across the whole world were collected. Our global dataset included 5753 potential phytopathogenic fungal species hypotheses from 20,312 samples distributed across all continents and major oceanic island regions. The samples and the corresponding metadata in GlobalFungi database are available in https://globalfungi.com/.
Sampling strategy	The collected samples must cover major terrestrial environments around the world. Given that the GlobalFungi database has integrated globally distributed samples from 178 original studies, the species-abundance data and the corresponding metadata of 19,669 samples were directly downloaded and used in our study. New samples were collected across East Asia from forest and cropland ecosystems, with samples from plant shoot, plant root, soil, and rhizosphere habitats during 2017-2021. Then, the published data in GlobalFungi and our new sequenced data were merged into a global dataset containing 20,312 samples.
Data collection	Samples distributed across cross all continents and major oceanic island regions, 11 land cover types (forests, grasslands, croplands, aquatic, deserts, woodlands, shrublands, tundra, wetlands, urban, and mangroves), and 12 habitat types (soils, plant shoots, roots, rhizosphere, deadwood, air, sediment, litter, lichen, freshwater, topsoil, and dust) were collected. The samples and the corresponding metadata in GlobalFungi database are available in https://globalfungi.com/, and the data in GlobalFungi database were contributed by hundreds of studies.
Timing and spatial scale	To synthesize a global atlas of phytopathogenic fungi, the samples collected from various periods across the globe were combined.<br>Newly generated data. Sample collection took place in 2017-2021. Continental Scale.<br>Meta-analysis data. Sample collection took place between 2000 and 2019. Global Scale.
Data exclusions	Sample with total sequences < 5000 were excluded.
Reproducibility	Information about the sampled locations and methods used in this paper are included in our method section. Almost all results in the

	current study could be reproduced using the data and methods we provided, except for those involving randomization, which may lead to some minor deviations.
Randomization	Given the potential unbalanced sampling effect, we randomly selected 300 or 150 samples in North America, Europe, Asia, Australia, South America, Antarctica, and Africa where >300 samples were collected. The diversity-latitude relationship based on subsampled data was fitted using both simple linear regression and second-order polynomial regression. Both random resampling procedures (300/150 samples) were conducted 100 times in R language, and the comparisons were also conducted 100 times to avoid the randomization bias.
Blinding	N/A

Did the study involve field work? ☐ Yes ☒ No

Reporting for specific materials, systems and methods

We require information from authors about some types of materials, experimental systems and methods used in many studies. Here, indicate whether each material, system or method listed is relevant to your study. If you are not sure if a list item applies to your research, read the appropriate section before selecting a response.

Materials & experimental systems

n/a	Involved in the study
☒	☐ Antibodies
☒	☐ Eukaryotic cell lines
☒	☐ Palaeontology and archaeology
☒	☐ Animals and other organisms
☒	☐ Clinical data
☒	☐ Dual use research of concern

Methods

n/a	Involved in the study
☒	☐ ChIP-seq
☒	☐ Flow cytometry
☒	☐ MRI-based neuroimaging
